# Interaction between Education and Household Wealth on the Risk of Obesity in Women in Egypt

**DOI:** 10.1371/journal.pone.0039507

**Published:** 2012-06-27

**Authors:** Amina Aitsi-Selmi, Tarani Chandola, Sharon Friel, Reza Nouraei, Martin J. Shipley, Michael G. Marmot

**Affiliations:** 1 Department of Epidemiology and Public Health, University College London, London, United Kingdom; 2 The Cathie Marsh Centre for Census and Survey Research, University of Manchester, Manchester, United Kingdom; 3 National Centre for Epidemiology and Population Health, The Australian National University, Canberra, Australia; 4 Department of Surgery, Imperial College Healthcare NHS Trust, London, United Kingdom; Tulane School of Public Health and Tropical Medicine, United States of America

## Abstract

**Background:**

Obesity is a growing problem in lower income countries particularly among women. There are few studies exploring individual socioeconomic status indicators in depth. This study examines the interaction of education and wealth in relation to obesity, hypothesising that education protects against the obesogenic effect of wealth.

**Methods:**

Four datasets of women of reproductive age from the Egyptian Demographic and Health Surveys spanning the period 1992–2008 are used to examine two distinct time periods: 1992/95 (N = 11097) and 2005/08 (N = 23178). The association in the two time periods between education level and household wealth in relation to the odds of being obese is examined, and the interaction between the two socioeconomic indicators investigated. Estimates are adjusted for age group and area of residence.

**Results:**

An interaction was found between the association of education and wealth with obesity in both time periods (*P*-value for interaction <0.001). For women with the lowest education level, moving up one wealth quintile was associated with a 78% increase in the odds of obesity in 1992/95 (OR; 95%CI: 1.78; 1.65,1.91) and a 33% increase in 2005/08 (OR; 95%CI: 1.33; 1.26,1.39). For women with the highest level of education, there was little evidence of an association between wealth and obesity (OR; 95%CI: 0.82; 0.57,1.16 in 1992/95 and 0.95; 0.84,1.08 in 2005/08). Obesity levels increased most in women who were in the no/primary education, poorest wealth quintile and rural groups (absolute difference in prevalence percentage points between the two time periods: 20.2, 20.1, and 21.3 respectively).

**Conclusion:**

In the present study, wealth appears to be a risk factor for obesity in women with lower education levels, while women with higher education are protected. The findings also suggest that a reversal in the social distribution of obesity risk is occurring which can be explained by the large increase in obesity levels in lower socioeconomic groups between the two time periods.

## Introduction

A worldwide obesity epidemic is unfolding [1]. The prevalence of a Body Mass Index (BMI) greater than thirty is increasing globally and at a much faster rate in low- and middle-income (henceforth, lower income) countries compared with high income countries [1], [2]. Obesity has been linked to several major chronic diseases, including type II diabetes, cardiovascular disease, selected cancers, gallbladder disease, asthma, osteoarthritis, and chronic back pain [3]. An estimated 2.6 million people die from non-communicable disease each year as a result of being overweight or obese, with the majority of deaths occurring in resource-poor countries [4].

Obesity has reached particularly alarming levels in the Middle East and North Africa (MENA) region [5], especially in Egypt – one of the most populous countries in the world [6]. By the 1990s, Egypt’s prevalence of female obesity (currently estimated at over 40% [7]) had already exceeded that in Europe and the USA [8]. At present, Egypt ranks among the top fifteen countries in the world in terms of age-adjusted diabetes prevalence (17%) [9] and this has worrying capacity and cost implications for future health services.

While obesity in emerging economies is increasingly recognised as a problem, the social distribution of obesity risk within these populations is disputed [10], [11]. Multi-country studies in Europe [12] and Latin America [8] support the hypothesis that obesity risk shifts from high to lower socioeconomic status (SES) groups as economic development proceeds [13]. They observe that in lower income countries obesity levels are greater among high socioeconomic groups (a high SES-high adiposity association), while in high income countries, like the US and Western Europe, obesity levels are greater among low socioeconomic groups (an inverse or low SES-high adiposity association). This was observed in women in particular [12], with a tipping point for the reversal in the association identified as a Gross National Product per capita of $2,500 [14]. More recent analyses of lower income country data present conflicting reports as to whether the SES-adiposity association is displaying the expected reversal [10], [11], [15].

However, partly due to data limitations, the majority of studies focus on a single SES indicator. Education level and household wealth are used interchangeably, despite the possibility that a woman’s education (usually beneficial for health [16]) may influence obesity risk in a very different way to the material wealth of the household she lives in. The few studies that have investigated the separate effects of these two factors on obesity in lower income countries document an obesogenic effect of wealth and a protective effect of education [17], [18]. This leaves epidemiology behind other disciplines in investigating the interplay between different socioeconomic risk factors like education and wealth on disease outcomes and fails to address the gap in research going beyond uni-dimensional analyses of socio-economic status and health [19].

In this study, using repeated cross sectional data over a period of rapid economic growth in Egypt, the interaction between education level and household wealth on obesity in women is investigated. As a hypothesis, we postulate that if wealth has an obesogenic effect, education might protect against this (education level might modify the association between wealth and obesity). Changes in the associations between education and wealth and obesity over time are also examined.

## Methods

### Data Set

The Demographic and Health Surveys (DHS) are a worldwide project funded by the United States Agency for International Development to provide data on demographics and health outcomes mainly for women and young children [20]. They are nationally representative household based cross-sectional surveys using a multistage stratified probabilistic sampling design (each primary sampling unit and household having a defined probability of selection) [20]. The DHS are a key source of data for studies on obesity [10], [11], [14], [21], [22], using extensive interviewer training, identical core questionnaires, standardised measurement tools and instrument pretesting to ensure standardisation across time and geographical locations [20].

### Study Sample

We use four waves of data in two distinct time periods. The data from 1992 and 1995 were pooled to create time period 1 (1992/95), and from 2005 and 2008 to create time period 2 (2005/08). The population eligible for interview and measurement was 49,948 women aged 15–49 years, but a small percentage of anthropometric data were not available for participants who were not at home (3% and <1% in time period 1 and 2 respectively) [20], resulting in an analytic sample of 49,058 women.

In the 1992 and 1995 surveys, only ever-married women with children under five years had anthropometric measurements taken. Therefore, women who had never been married and had not given birth in the preceding five years were excluded from the 2005/2008 period (N = 11,852) to ensure comparability of the two time periods. Women who were pregnant were excluded from both samples (N = 1,821), as well as 1,207 women who were missing covariates, leaving a total of 34,178 women in the final analytic sample (11,097 in the 1992/95 sample and 23178 in the 2005/08 sample). Detailed sampling plans are available from the final report of the surveys. Response rates were >95% across all survey waves [20].

### Outcome and Independent Variable Definitions

A BMI cutoff outcome is used as this remains the most widely available measure for studying weight status in populations. Obesity is defined as a Body Mass Index ≥30 [7] and calculated as (weight/height^2^). Weight was obtained using digital scales designed and manufactured under the authority of the UN children’s fund. Height was obtained using measuring boards produced by Shorr Productions for use in survey settings [20].

Education level was coded into three categories (1 = no or primary education, 2 =  secondary education, 3 =  higher education), based on prior research suggesting that variation in obesity levels in women is greatest when comparing higher levels of education with no or primary education [8]. The wealth index is based on amenities, assets, and housing conditions. It was generated through a principal components analysis, using the Filmer & Pritchett method to calculate factor loadings and derive a score for each household [23]. The wealth index was generated separately for each survey year and the quintile cut-offs based on the weighted distribution of the household population for each specific year. Area of residence was included as a simple adjustment for environmental exposures. This included two categories: urban (cities >50,000 inhabitants and towns) and rural (≤50,000 inhabitants). We include age groups in ten year age bands as a biological confounder. To simplify the presentation of subgroup frequencies in **Tables 1** **and** **2**, wealth quintiles were combined to create a poorer group (quintiles 1 and 2) and a richer group (quintiles 4 and 5) with quintile 3 coded as missing. This did not affect the overall pattern observed when all quintiles were used. We also created a variable called year to represent each of the two time periods (1992/95 = 1; 2005/08 = 2).

**Table 1:** Sample characteristics – Egyptian DHS 1992/95 and 2005/08.

	1992/95		2005/08	
	N = 11097		N = 23178	
	Total N	% (SE)	Total N	% (SE)
BMI (kg/m^2^)				
Non-obese (BMI<30)	8680	78.2 (0.8)	14123	60.9 (0.5)
Obese BMI (≥30)	2416	21.8 (0.8)	9055	39.1 (0.5)
Total	11097		23178	
Education				
None/primary	7550	68.0 (1.1)	10495	45.3 (0.7)
Secondary	2938	26.5 (0.8)	10174	43.9 (0.6)
Higher	609	5.5 (0.5)	2509	10.8 (0.4)
Total	11097		23178	
Area of residence				
Urban	4669	42.1 (2.2)	9422	40.7 (1.4)
Rural	6427	57.9 (2.2)	13756	59.4 (1.4)
Total	11097		23178	
Age group (years)				
15–24	2579	23.2 (0.6)	4177	18.0 (0.3)
25–34	5725	51.6 (0.6)	9919	42.8 (0.4)
35–49	2792	25.2 (0.5)	9081	39.2 (0.4)
Total	11097		23178	
**Wealth by education level**				
None/primary				
Poorer 40%	4701	78.5 (1.6)	6545	78.8 (0.9)
Richer 40%	1286	21.5 (1.6)	1761	21.2 (0.9)
Total	5987		8306	
Secondary				
Poorer 40%	462	20.5 (1.7)	2250	29.2 (1.0)
Richer 40%	1792	79.5 (1.7)	5447	70.8 (1.0)
Total	2254		7696	
Higher				
Poorer 40%	3	0.3 (0.2)	81	3.5 (0.5)
Richer 40%	568	99.7 (0.2)	2244	96.5 (0.5)
Total	570		2326	

**Table 2:** Prevalence of obesity by subgroups – Egyptian DHS 1992/95 and 2005/08.

	1992/95		2005/08		Absolute difference^a^	
	N = 11907		N = 23177			
	N obese	% (SE)	N obese	% (SE)	% (SE^b^)	P-value^c^
Education						
None/primary	1439	19.1 (0.8)	4124	39.3 (0.7)	20.2 (1.1)	<0.0001
Secondary	780	26.6 (1.2)	3910	38.4 (0.7)	11.9 (1.4)	<0.0001
Higher	198	32.5 (2.3)	1020	40.7 (1.3)	8.2 (2.7)	0.004
Total	2416		9055			
Wealth quintile						
Poorest 20%	205	7.8 (0.6)	1210	27.9 (0.9)	20.1 (1.1)	<0.0001
Poorer 20%	360	14.2 (0.9)	1555	34.3 (0.9)	20.1 (1.3)	<0.0001
Middle 20%	502	22.0 (1.0)	1966	40.5 (1.0)	18.5 (1.4)	<0.0001
Richer 20%	667	33.9 (1.4)	2202	45.3 (1.0)	11.4 (1.7)	<0.0001
Richest 20%	681	40.6 (1.6)	2121	46.2 (1.1)	5.7 (1.9)	0.003
Total	2414		9055			
Area of residence						
Urban	1519	32.5 (1.2)	4208	44.7 (0.8)	12.1 (1.4)	<0.0001
Rural	897	14.0 (0.7)	4847	35.2 (0.7)	21.3 (1.0)	<0.0001
Total	2416		9055			
Age group						
15–24	280	10.8 (0.9)	782	18.7 (0.8)	7.9 (1.2)	<0.0001
25–34	1255	21.9 (0.9)	3372	34.0 (0.7)	12.1 (1.1)	<0.0001
35–49	881	31.6 (1.3)	4901	54.0 (0.7)	22.4 (1.5)	<0.0001
Total	2416		9055			
**Wealth groups by education level**						
None/primary						
Poorer 40%	513	10.9 (0.6)	2175	33.2 (0.8)	22.3 (1.0)	<0.0001
Richer 40%	541	42.1 (1.7)	948	53.9 (1.5)	11.8 (2.3)	<0.0001
Total	1054		3123			
Secondary						
Poorer 40%	52	11.3 (1.9)	564	25.1 (1.1)	13.8 (2.1)	<0.0001
Richer 40%	622	34.7 (1.5)	2457	45.1 (0.9)	10.4 (1.8)	<0.0001
Total	674		3021			
Higher						
Poorer 40%	0	d	27	32.9 (5.3)	–	–
Richer 40%	184	32.4 (2.4)	918	40.9 (1.4)	8.5 (2.8)	0.004
Total	184		945			

a Difference in prevalence between the two time periods (prevalence_(2005/08)_ – prevalence_(1992/95)_).

b SE calculated as square root of ((SE_2005/08)_^2^ + (SE_1992/95_)^2^).

c Based on the chi-squared test for the difference in prevalence.

d There are only three women in this group so no estimate of prevalence is given.

### Statistical Analysis

STATA 12 SE® survey commands (svy) were used to analyse the data and account for the complex design effect. These commands take into account the effect of clustering and unequal weights as appropriate when computing frequencies, proportions, variance, standard errors, and confidence intervals. Sampling characteristics and the prevalence of obesity by subgroup were computed in both periods, using a chi-squared test to compare differences between periods. The absolute difference in prevalence between the two time periods was calculated as prevalence_(2005/08)_ – prevalence_(1992/95)_ for each subgroup. Logistic regression was used to analyse the association between the SES indicators and obesity (both unadjusted and adjusted for age group and area of residence).

To examine the main hypothesis of whether education level modified the association between wealth and obesity, interaction terms between education and wealth were fitted in the logistic regression models. The interaction terms were examined for significance using the Wald test (the null hypothesis being that the interaction terms in the regression model are equal to zero). We use the Wald test rather than a likelihood ratio (LR) test because the latter is not valid with estimation procedures that adjust for the design effect in STATA®. However, we performed a sensitivity analysis using LR tests without adjusting for the design effect to examine whether the data were better fitted using a model with or without the interaction. In preliminary analyses, it became apparent that the time period variable was a strong effect modifier of the SES–obesity relationship. The *P*-values for the interaction terms between each of education level and wealth quintile and the time period variable were highly significant (*P*<0.0001 in all models for both unadjusted and adjusted estimates). On this basis, we proceeded in performing the analyses separately for each of the two time periods.

We estimated the unadjusted odds ratio (OR) between each SES indicator and obesity, as well as the OR adjusted for age group and area of residence in each time period, to examine how the associations changed over time. The interaction results were compiled based on recommendations by Knol et al. to present both the separate and joint effects of two interacting factors [24]. First, the wealth trend (effect of an increase in one wealth quintile) was estimated in the no/primary education group using the wealth quintile variable in its continuous form. Then, using the interaction terms for wealth by education, we calculated the wealth trend among the two higher education groups and tested the hypothesis that education level modifies the effect of wealth. The interaction was fully illustrated in a graph, plotting the log odds of obesity calculated for each combination of education level and wealth quintile using the category ‘education level  =  none/primary and wealth quintile  =  poorest’ as the referent. This estimation was performed using logistic regression by fitting dummy variables to indicate the combinations of education level and wealth quintile, to produce the log ORs adjusted for age group and area of residence. Potential multicollinearity between the co-variates used in the multivariate regression model was assessed using variance inflation factors.

### Ethical Review

Demographic and Health Surveys (DHS) data collection procedures were approved by the Measure DHS (Demographic and Health Surveys) (Calverton, MC) Institutional Review Board and by the national body that approves research studies on human studies in Egypt. Written consent was obtained by the interviewers from each participant. The use of the Egyptian data for this particular study was approved by Measure DHS, and considered exempt from full review by University College London because the study is based on an anonymous, public-use data set with no identifiable information on the survey participants.

## Results

The average age of women in the DHS survey was 29.3 years (95%CI: 29.1, 29.5) in the 1992/95 sample and 33.1 years (95%CI: 33.0, 33.3) in the 2005/08 sample. The overall prevalence of obesity was 21.8% (95%CI: 20.2, 23.3) in the 1992/95 sample and 39.1% (95%CI: 38.0, 40.1) in the 2005/08 sample. Over 90% of the women in the sample were married.

### Sociodemographic Characteristics and Obesity Prevalence

**Table 1** shows that the women in the 2005/08 sample were more educated, with similar proportions residing in urban and rural areas in both time periods. The distribution of wealth by education group also remained very similar over time with wealth being concentrated among the more highly educated. Spearman rank correlation coefficients between education and wealth were 0.56 and 0.57 in the 1992/95 sample and the 2005/08 sample respectively.

### Trends in Obesity Prevalence

**Table 2** shows prevalence trends in obesity. The prevalence was significantly greater in all subgroups in the 2005/08 sample. The largest absolute increase in prevalence over time occurred in those who were less educated, poorer and living in rural areas (absolute increase in prevalence percentage points of 20.2, 20.1, and 21.3 in women with no/primary education, poorest wealth and rural residence respectively). There was an appreciably lower increase in those who were more educated, richer and urban dwelling (absolute increase 8.2%, 5.7% and 12.1% in the higher educated, richest, and urban groups respectively). For the 1992/95 time period, there were only three women with higher education who were in the two poorest wealth quintiles, therefore no prevalence calculation was made involving these groups.

### Changing Association of Education and Wealth with Obesity, and Interaction Results

The top half of **Table 3** shows the relationship between the SES indicators and obesity defined as a binary outcome, displaying separate effects for education and wealth (unadjusted and adjusted for age group and area of residence). There was a positive association between age group and obesity as expected (not shown but results available on request). The unadjusted ORs indicated a positive association between each of education and wealth and obesity in the 1992/95 sample. The odds of obesity in the higher education group compared with the group with no or primary education were 2.04 times higher (95%CI: 1.62, 2.58), and the odds of being obese increased by 1.68 times for each increase of one wealth quintile (95%CI: 1.61, 1.76). Adjustment for age group and area of residence diminished the magnitude of the estimates for education (OR; 95%CI: 1.23; 0.94, 1.19 for the higher education group relative to the group with no or primary education) as well as for wealth (1.57; 1.48, 1.77). In the 2005/08 sample there was little evidence of an association between education and obesity (see table 3), and the positive association between wealth and obesity was diminished in magnitude compared with the earlier period (OR; 95%CI: 1.22; 1.19, 1.26).

**Table 3:** Separate and joint effects of education and wealth on obesity. Egyptian DHS 1992/95 and 2005/08.

	1992/95 (N = 11907)					2005/08 (N = 23177)			
	Unadjusted			Age and area of residence adjusted		Unadjusted		Age and area of residence adjusted	
	OR		95%CI	OR	95%CI	OR	95%CI	OR	95%CI
**Separate effects**									
Education (level)									
None/primary	1			1		1		1	
Secondary	1.54		(1.35–1.75)	1.39	(1.20–1.60)	0.96	(0.90–1.04)	1.18	(1.09–1.27)
Higher	2.04		(1.62–2.58)	1.23	(0.94–1.60)	1.06	(0.94–1.19)	1.02	(0.90–1.16)
Wealth quintile (linear)	1.68		(1.61–1.76)	1.57	(1.48–1.67)	1.22	(1.19–1.26)	1.21	(1.17–1.25)
**Joint effects (wealth trend within education levels)** a									
Education (level)									
None/primary				1.78	(1.65–1.91)			1.33	(1.26–1.39)
Secondary				1.50	(1.35–1.68)			1.21	(1.15–1.27)
Higher				0.82	(0.57–1.16)			0.95	(0.84–1.08)
*P*-value^b^				<0.001				<0.001	

a Odds ratio of obesity associated with an increase in one wealth quintile in each education group estimated from the model including an interaction between education and wealth (estimates adjusted for age group and area of residence).

b *P*-value from the LR test for an interaction between education level and wealth quintile in its continuous form.

**Figure 1:**
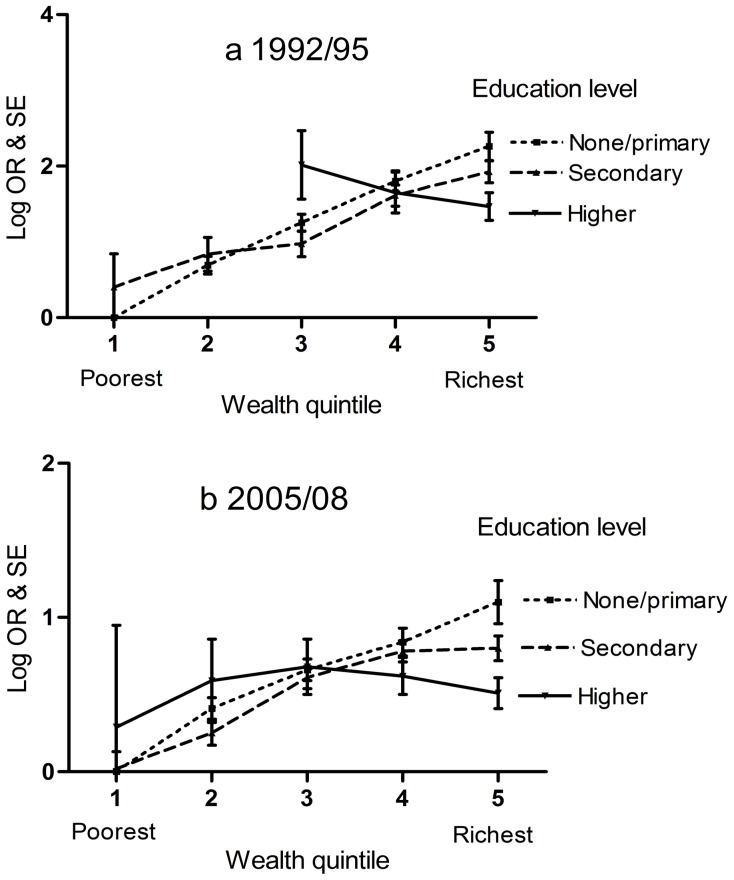
Interaction between women’s education level and household wealth on obesity risk using the Egyptian DHS a. 1992/95; b. 2005/08. Each point represents the log OR of that combination of education level and wealth quintile compared with the reference category (education level  =  none/primary and wealth quintile  =  poorest). Error bars represent the standard error of the log OR. All plotted estimates are adjusted for age group and area of residence.

**Figure 1** plots the log OR for obesity for each combination of education and wealth quintile. No points are plotted in 1992/95 for the two poorest groups in the higher education category as there were only three women in these groups (see table 1). The pattern appears to be consistent in both time periods and shows a positive association between wealth and obesity in the group with no/primary education and a similar but diminished positive association in the group with secondary education. The group with higher education shows a much shallower relationship of obesity with wealth quintile. At the higher end of the wealth distribution (quintile 5 on the x-axis) education appears to have a protective effect (a high education-low obesity association – *P* for trend <0.001). There is little evidence of an association between education and obesity in the other wealth quintiles (*P* for trend not significant). The figure also shows that the effects of wealth quintile on obesity are well described by fitting wealth as a continuous variable.

The lower part of **Table 3** displays the results for the regression model including the interaction term between education level and wealth quintile in its continuous form. The Wald test supported the presence of an interaction (*P*≤0.01 for all interaction terms in both periods). The LR test for whether the model fit was better with or without an interaction between education level and the linear wealth quintile variable also provided strong evidence of an interaction (*P*<0.0001 in both periods). The variance inflation factors for the covariates ranged between 1.0–2.5 in both the 1992/95 and 2005/08 samples indicating multicollinearity was not a concern.

The joint effect estimates show a strong positive association between wealth and obesity in the no/primary education group and the secondary education group, but no association in the group with higher education. In the earlier sample (1992/95) moving up one wealth quintile was associated with a 78% increase in the odds of obesity (OR; 95%CI: 1.78; 1.65, 1.91) for women with the lowest level of education (none/primary). For women with the highest level of education, there was little evidence of an association: the OR suggested a marginal reduction in the odds of obesity for the same increase in wealth of 18% that was not statistically significant (OR; 95%CI: 0.82; 0.57, 1.16). The results for the 2005/08 sample were similar but attenuated in the lowest level of education (none/primary): moving up one wealth quintile was associated with a 33% increase in the odds of obesity (OR; 95% CI: 1.33; 1.26, 1.39). In the highest level of education, there was again little evidence of an association between wealth and obesity (OR; 95%CI: 0.95; 0.84, 1.08).

### Sensitivity Analyses

The analyses were repeated in the 2005/08 sample to include all ever-married women (not just those with under-5 year old children). This showed a similar interaction pattern between education and wealth which was statistically significant, suggesting the results of this analysis are generalisable beyond women with young children. The interaction results were reproducible in the separate survey years (LR test *P*<0.001 in 1992, 2005 and 2008; LR test *P* = 0.05 in 1995). We also performed a sensitivity analysis incorporating the number of children born to the respondent as a variable but this had little impact on the estimates and did not improve the model fit.

Some authors have suggested that logistic regression is unsuitable for use when the outcome prevalence is common (>10%), proposing a modified Poisson or log binomial model instead [25]. In this particular study, logistic regression was deemed the most appropriate method for a number of reasons including the cross-sectional nature of the data, and the ease of comparison with other studies [26]. However, we ran the model using the modified Poisson and log binomial models and found the interaction pattern (the direction of the associations and their statistical significance) was preserved.

## Discussion

This study reports that education level modifies the association between household wealth and obesity in two nationally representative samples of women in Egypt. In women with the lowest education level (no/primary education), moving up one wealth quintile is associated with a 33 to 78% increase in the odds of obesity depending on the time period. In contrast, for women with the highest level of education there was little evidence of a significant association. Secondary findings are: 1) a faster rate of increase in obesity prevalence among the poorer, less educated and rural groups; and 2) a diminishment of the positive association between each of education and wealth and obesity over time.

### Comparison with Prior Studies

The sample composition in this study is consistent with national estimates from other sources which also show stable proportions of urban and rural dwellers, as documented in the Egyptian national census [20] and investigated elsewhere [27]. Similarly, the proportion of women reporting that they are not in paid employment in our sample was over 70%, which is consistent with the relatively low labour participation rates (<30%) reported in Egyptian women over 15 years of age during the time period of 1992–2008 [6].

Since the early 1990s, there has been a growing number of studies using data from lower income countries to examine the association between single SES indicators and adiposity [8], [13], [14], [21] and how it changes over time [10], [12], [28]. The majority of these studies support the hypothesis of a reversal of the social gradient of obesity, and some provide evidence that lower SES groups are experiencing a rapid increase in obesity risk [10], [12], [28]. The diminishing magnitude of the estimates for education and wealth found in this study is consistent with this body of research, including the predicted tipping point for the reversal (a GNP per capita of $2500 [14]) - Egypt’s GNP per capita grew from ∼$600 to $1,801 over the examined period [6]. Based on this, the high SES-high obesity association in Egyptian women should therefore be expected to continue diminishing in magnitude, and eventually reverse in the next few years to more closely match the inverse (high SES-low adiposity) association found in high income countries [13], [14]. The findings suggest that this is mainly driven by a large increase in the prevalence of obesity in lower SES and rural groups, relative to a much smaller increase in the higher SES and urban groups.

When examined jointly using the interaction model, education and wealth appear to influence obesity levels differently in Egypt. This could be explained away in terms of the validity and reliability of education and wealth as indicators of SES [11], however other lower income countries have reported separate effects of education and wealth including Peru, the Philippines, China, and Brazil [17], [18], [29], [30]. They show a consistently positive association between wealth and obesity and an inverse association between education and obesity. These patterns are comparable to earlier findings from Eastern Europe during its economic transition in the early 1990s when education and material circumstances acted differently as SES indicators of health outcomes [28].

There is a limited literature to compare the interaction results with, as few studies investigate the relationship between different SES indicators of health [29], [31]. One study examining the interaction between maternal education and wealth in relation to childhood infection in Latin American found a comparable pattern: for any given level of wealth, the children of educated mothers were healthier compared with the children of less educated mothers [31]. Another, found a protective effect of education against overweight/obesity among women but not men [29]. We propose that using a single indicator to represent SES may fail to capture the complexity of the social distribution of obesity in lower income countries, and does not give education and wealth appropriate consideration as important factors in their own right on the causal pathway of obesity. Therefore, the relationship between different SES indicators and excess adiposity requires further investigation in rapidly changing economies.

### Plausible Mechanisms

The findings from this analysis support the hypothesis that education might protect against the obesogenic effects of wealth. A simple explanation for these findings may be that increasing wealth in lower income countries results in greater access to food, an escape from physical labour [32], and therefore a higher risk of obesity. For example, increased wealth or income in lower income countries is thought to promote poor dietary habits involving take-away food and fast-food [33] and the effect may be worse among less wealthy households due to a link with food insecurity [34]. Higher education level, on the other hand, may protect against the obesogenic effect of wealth by positively informing health behaviours including dietary choices [16].

This explanation is supported by data from Mexican and Colombian cash transfer programs which show that cash accumulation among the poor increases consumption of fat and sugar, especially when adult education sessions are not a compulsory part of the program [35], [36]. The beneficial effect of education has been theorised in the psychology literature as resulting from a set of cognitive abilities that directly influence health decisions as well as enabling access to other forms of knowledge and understanding which promote health [16], alongside psychosocial pathways [37].

It is likely that there are other factors associated with education that may influence the obesogenic effect of wealth. It was not possible to take these into account in this study, but cultural preferences affecting body shape norms and attitudes towards physical activity may have a role [13]. Since education is not a guaranteed way out of poverty in Egypt [38], some women may need to forego education and seek upward mobility through other routes such as marriage, thus creating a stratum of women who have better living conditions but low education levels. These women are likely to come from more traditional backgrounds which may impose restrictions on their mobility and favour higher body weight as a beauty standard. In contrast, wealthy women with high levels of education may come from more progressive families, and adopt more Western norms of female beauty that favour slim body shapes as well as endorse more physically active leisure time activities. At the higher end of the wealth distribution, women may be able to more to fully reap the benefits of higher education to enable healthier behaviours through access to expensive foods and sports facilities. For a more complete understanding of the interaction between education and wealth contextual factors such as changes in the food environment as well as locally relevant factors would need to be taken into account.

The excessively high overall prevalence of female obesity in Egypt relative to its level of economic development is currently unexplained, although other investigations have discussed the role of the food subsidy system in Egypt which makes oil, sugar, and previously ghee available more cheaply [39], [40]. In addition, there may be a biology-environment interaction specific to countries where the nutrition transition (from a situation of calorie scarcity to one of high energy density) has been rapid compared with Western countries. The rapid change experienced within individual lifespans could have caused a mismatch between metabolic programming in early life and the energy available for consumption in adulthood, with a consequent increase in the susceptibility to excess adiposity, as formulated in Gluckman’s match-mismatch hypothesis [41].

The remarkably large increase observed in lower SES groups in this study may be a result of the rapid adoption of new patterns of consumption (diets high in sugar and saturated fat) and sedentary levels of physical activity in these groups [4]. For example, the consumption of soft drinks in Egypt has increased dramatically over the period examined, particularly in groups of lower SES status [42], [43]. There are few empirical studies of SES and dietary patterns in Egypt in the literature, however, one study reported a greater propensity to consume meat, fats, and sugar in the poorer rural populations than in urban ones [44]. The faster rate of increase in obesity levels in the lower SES groups has been attributed elsewhere to a disproportionate response to the contextual changes of the nutrition transition [10] and again may be linked to a mismatch between metabolic programming and energy availability [41].

### Implications for Intervention

While exercising due caution in making policy suggestions based on findings from cross-sectional data, the health benefits of women’s education are well documented in the fields of maternal and child health [31]. This paper shows how the education of women can also protect against non-communicable disease risk factors such as obesity, and therefore that potential synergies in different health fields may exist. We suggest that the association between economic development and higher obesity levels in women is not inevitable. It may be broken by investment in education programs alongside programs promoting economic growth. The findings also caution against the unintended consequences of poverty alleviation programs that do not do so.

### Strengths and Limitations

This analysis took advantage of the opportunity offered by the Egyptian DHS data, which are unique in the number of repeat standard survey waves, to create two samples of pooled data. This provided large sample sizes and hence greater power to reveal underlying trends and associations which may otherwise be obscured by noise or large sampling variability in smaller individual data sets. The data limitations reduced the sample to ever-married women with children under-5 which may limit the generalisability of the findings, however, the high marriage rate in Egypt (estimated at >80% in the 20–49 years age group [45]) and the sensitivity analysis suggest the patterns observed are likely to be found in the general population of Egyptian women in the reproductive age range.

Importantly, this study goes beyond the uni-dimensional analyses of SES and obesity in order to separate the associations between individual SES indicators and obesity and examine how they intersect. However, as in all studies using cross-sectional data, all the co-variates were measured at the same time and the results can only support the original hypothesis, although theory and prior studies provide substantial corroborating evidence for the postulated protective effect of education.

### Conclusion

In summary, wealth appears to be positively related to obesity in women in Egypt as in many other lower income countries, but education may offer some protection against this - particularly higher education. To our knowledge, this is the first time this interaction has been demonstrated in any population. In addition, the study supports the existence of a dynamic association between each of education and household wealth in relation to obesity over time, consistent with expectations from the majority of prior studies documenting a reversal of the association between SES and obesity as economic development proceeds. Compared with higher income countries, the relationship between SES and body size appears to be more complex in lower income countries undergoing rapid economic growth. We recommend that future studies on SES and excess adiposity in these rapidly changing settings consider the relationship between SES indicators with greater scrutiny. Finally, the remarkable increase in obesity prevalence among the less educated, poorer and rural dwelling women requires further investigation.
